# Commentary on “Periprosthetic Fluid Analysis in the Diagnosis of Breast Implant Infections Using Cell Count and Differential”

**DOI:** 10.1093/asjof/ojaa034

**Published:** 2020-07-07

**Authors:** Peter W Thompson

I wish to congratulate the authors on their well-written and impactful report entitled “Periprosthetic Fluid Analysis in the Diagnosis of Breast Implant Infections Using Cell Count and Differential.” ^1^ Herein, the authors conclude that the percentage of polymorphonuclear (PMN%) lymphocytes present in the periprosthetic fluid can inform decision making in cases of suspected implant infection following breast reconstruction.

Few problems faced by plastic surgeons and our patients are as devastating as infection following implant-based breast reconstruction. Infectious complications following the placement of a breast implant or tissue expander lead to decreased patient satisfaction with their reconstruction,^2^ increased rates of reconstructive failure,^3^ and importantly increased expense for the patient and hospital system. Some estimates put this cost increase at more than $12,500 per event.^4^ Estimates of the incidence of infectious complications following implant reconstruction are as high as 35%.^5^ Despite the potential for severe physical/psychological morbidity to the patient and a cost to the healthcare system of slightly less than the price of a new mid-size sedan, there exists a surprising paucity of data regarding objective measures to guide decision making and predict the outcomes in patients with suspected implant infections.^6^ As a specialty, what we need is a noninvasive tool, both sensitive and specific, that can sort patients easily into 4 groups: those without infection, those with infection who will improve with antibiotics alone, those with infection whose reconstruction can be salvaged with surgery, and those who require explantation. With the data reported in this paper, the authors have taken an important step toward uncovering this diagnostic “holy grail.”

In this retrospective review, the authors have analyzed the cell counts and differentials of periprosthetic fluid collections obtained from 44 patients with suspected implant infections. The most important finding was that a PMN% cutoff value of 77% was able to differentiate between patients with and without prosthetic infection with a sensitivity of 89% and specificity of 93%. The authors also found that higher PMN% was also correlated with outcome, predicting the need for surgery and explantation. Interestingly, different causative microorganisms were associated with significantly different average PMN%.

This report has several limitations worth discussion, apart from those which derive from the retrospective nature of this analysis. First, one may take issue with the authors’ definition of “prosthetic infection.” All patients included in this analysis had some clinical sign suggestive of infection, such as fever, breast erythema, or swelling; however, a patient was considered to have a periprosthetic infection only when positive cultures were obtained from periprosthetic fluid, or, somewhat subjectively, when intraoperative findings were suggestive of infection. This methodology excludes 2 important groups: those who had a true prosthetic infection but were culture-negative due to successful treatment/sterilization of fluid from antibiotic therapy and those who had a true prosthetic infection but were not included in the analysis because they lacked a periprosthetic fluid collection to sample. For example, it is common for patients with surgical drains to develop a periprosthetic infection, but it is less common for patients with surgical drains to develop large, clinically significant periprosthetic fluid collections. Removal of these 2 groups from the analysis may have skewed the analysis in favor of patients with more severe, recalcitrant infections. In practice, any patient with clinical signs of infection and a periprosthetic fluid collection will likely be treated as a periprosthetic infection regardless of laboratory findings or culture results; therefore, it may be appropriate to state that the true decision-guiding power of PMN% is to discern between more severe and less severe implant infections.

Second, this analysis lacks data from an important control group: PMN% from patients with periprosthetic fluid collections and no clinical evidence of infection. Seroma or persistently high drain output following implant-based breast reconstruction has an overall incidence of around 5.4%.^7^ There is evidence that seromas represent one end of an inflammatory spectrum that left untreated may eventually progress to a periprosthetic infection. If PMN% measured from periprosthetic fluid in patients before any clinical or laboratory sign of infection were found to be the lowest of all the sampled groups, it would lend validity to PMN% as an analytic tool and establish an important baseline for comparison.

Third, the authors suggest that not all pathogenic microorganisms result in the same PMN% increase. For example, while *Group A streptococcus* resulted in a PMN% of 97%, *Propionibacterium acnes* resulted in a PMN% of 19%, well below the suggested 77% threshold. While it is true that *P. acnes* is in general a much less common pathogen than other gram-positive bacteria, such as *Staph* and *Strep* species, the sensitivity of PMN% as a decision-making tool may depend significantly on the pathogenic “biogram” at a given hospital or facility. 

The principle supposition presented in this paper, that periprosthetic fluid PMN% greater than 77% is predictive of implant infection, has the potential to impact the clinical practice of any plastic surgeon who performs breast reconstruction. However, the ability to discern between patients with and without implant infection is rarely the dilemma. Given the relatively high incidence of post-reconstruction implant infection, clinical suspicion is usually high and threshold to treat very low. The more pressing question is, once a patient has been diagnosed with a periprosthetic infection, how should they be managed? At our institution, the decision to treat with antibiotics alone, salvage the implant, or perform explantation depends on a myriad of factors. These include clinical judgment of infection severity, potential for disruption of adjuvant therapy, and not least of all patient desires. The authors propose that PMN% also correlates with the outcome, showing that patients managed operatively had significantly higher PMN% than those managed nonoperatively; however, PMN% less clearly discerned patients who underwent implant salvage from those requiring explantation. As the authors are surely aware, a more detailed application of this measure in a prospective fashion is necessary to determine its true validity. Another avenue of exploration would be trends in periprosthetic PMN% for individual patients over the course of treatment. Would a drop in periprosthetic fluid inflammatory markers following treatment with antibiotics during a single hospitalization predict better patient outcomes?

In conclusion, the data presented in this report represent a step forward in our understanding of the pathology of periprosthetic infections. Additional study is needed in order to determine if this information can truly help us counsel patients and make difficult decisions about implant salvage. When it comes to breast implant infection, prevention is the best medicine; however, until our rate of infection is zero, plastic surgeons and our patients will continue to make difficult treatment decisions, and we will continue our search for the “holy grail.”
